# Editorial

**Published:** 1844-02-10

**Authors:** 


THE MEDICAL EXAMINER.

PHILADELPHIA, FEBRUARY 10, 1844.

### YALE COLLEGE.

The catalogue for the present year, of this old and respectable institution, exhibits it in its wonted prosperity.

The number of the Medical Class is sixty. Of these, it appears by the New Haven Herald of the 24th ultimo, that *eighteen* were admitted to the Degree of Doctor of Medicine at the late Commencement.

### SYDENHAM SOCIETY.

We understand that a society has been formed in London with the above title, consisting of many of the most distinguished members of the medical profession, the leading object of which is to meet certain acknowledged deficiencies in existing means for diffusing medical literature, which are not likely to be supplied by the efforts of individuals. "The Society will carry its objects into effect by a succession of publications, embracing, among others: 1, Reprints of standard English works, which are rare or expensive; 2, Miscellaneous selections, from the ancient and from the earlier modern authors, reprinted or translated; 3, Digests of the works of old and voluminous authors, British and foreign, with occasional biographical and bibliographical notices; 4, Translations of the Greek and Latin medical authors, and of works in the Arabic and other eastern tongues, accompanied, when it is thought advisable, by the original text; 5, Translations of recent foreign works of merit, and orginal works of merit, which might prove valuable as books of reference, but which would not otherwise be published, from the slender chance of their meeting with a remunerating sale, such as bibliographies, alphabetical and digested indexes of voluminous periodical publications, &c.

Already, the list of members amounts to upwards of 1400; and the Society feels itself authorized to take immediate steps for carrying its intentions into effect. Two works are in press, which are expected to appear very shortly. The subscription constituting a member is *five dollars*, to be paid in advance, in March, annually, for which he is entitled to a copy of every work published by the Society for the year for which he subscribed.

The works of the Society will be handsomely printed, on a uniform plan, for members only. It is not competent for book clubs to subscribe to the Society as members, but it will be permitted to societies and institutions, having *permanent* libraries, to subscribe to the Society, in the name of their President, Secretary or Librarian, or other responsible officer."

The Society invites the co-operation of members of the profession in the United States, and, to enable such to participate in the enterprise, has requested Professor Dunglison to act as Local Honorary Secretary for Philadelphia, which he has consented to do. To him the published works of the Society will be forwarded to subscribers in this country, and subscriptions may be sent through the same channel.

"*Case of successful Peritoneal Section for the removal of two diseased Ovaria complicated with Ascites.* By JOHN L. ATLEE, M. D., of Lancaster city, Penn.

This is the title of a most valuable paper contained in the last number of "The American Journal of the Medical Sciences," and referred to by Professor Dunglison in his communication inserted in the last number of the Examiner.

The patient was about twenty-five years of age, and had been in bad health since December 1836. On the 2d of June, 1840, she was tapped, and twenty-pounds of a clear and very light straw-coloured serum were removed. In July, 1842, twelve-pounds more were taken away; in December of the same year, twenty-two pounds. At this time, during the escape of the fluid, Dr. W. L. Atlee "was sensible of a hard substance falling against his hand; which, upon examination, proved to be a rounded hard tumour just above the right inguinal region, rising out of that side of the brim of the pelvis, and as large as a turkey's egg. It was movable to some extent, but seemed attached to the right side. After a careful examination the narrator and his brother, Dr. W. L. Atlee, came to the conclusion that the tumour originated in the right ovarium; and, from the absence of all other evidence, they believed, had been the cause of the ascites. From this time the tumour was frequently examined, and was observed gradually to increase. On the 13th of May last (1843,) thirty-two pounds of serum were removed, possessing all the characteristics of the fluid previously taken away, except that it was rather more highly coloured. The tumour was found to have rapidly increased, and was now quite as large above the brim of the pelvis as the fœtal head."

"*Operation.*—June 29, 1843. The patient was placed upon a common dining table with the leaves down. Her body was slightly elevated, with her head and shoulders supported by pillows, her feet resting on chairs about two feet apart. Standing on her right side, with my brother, Dr. W. L. Atlee, as my principal assistant, and in the presence of Drs. Humes, Parry, Neff, and Ehler, and of Messrs. Bare, Coxe, and Richards, our pupils, all of whom promptly and kindly rendered such assistance at the several stages of the operation, and at such stations about the patient as had been previously assigned to them, I commenced by making an incision about nine inches long, through the skin and cellular tissue, from half an inch below the umbilicus to within an inch and a half of the upper surface of the symphysis pubis. The length of the incision between these two points was owing to the ascitic distension. An opening was now carefully made through the linea alba, a director introduced, and an incision made for an inch each way above and below the opening. Some slight difficulty occurred in doing this exactly in the median line, owing to the adhesions between the parts, occasioned by the previous wounds in tapping. The peritoneum was now exposed, and to test the fact, whether the dropsy were peritoneal or encysted, which had been the subject of discussion between my brother and myself, a very small opening was made through it with the point of the scalpel. This was immediately followed by a discharge of thin and pale straw-coloured serum. Being now satisfied that the dropsy was peritoneal, and had been caused by the obstruction to the circulation, and irritation produced by the tumours, I plunged a trocar through the opening, and drew off about eighteen pounds of a similar fluid. The abdominal cavity was then emptied as much as possible, by pressure on its whole anterior surface, and the fascia and peritoneum opened by the probe-pointed bistoury, guided first by the director and subsequently by the two first fingers, to the extent of the first incision. About two pounds more of serum escaped from the most dependent part of the wound. The first thing that presented itself, when the cavity of the abdomen was opened, was the upper and inner part of the tumour, as large as a goose-egg, composed of very small hydatids, varying from the size of a millet seed to that of a dried pea, and of a cream colour, projecting out of the pelvis on the right side, above the peritoneal investment of the tumour, and overlapping it like a mushroom for half an inch in every direction. Above this, and to the right, was seen that portion of the tumour which projected highest above the brim, covered by peritoneum, and extending over the iliac vessels. In the centre of the pelvis, and very much 'in situ,' the fundus uteri could be seen, forming the plane surface previously felt through the integuments, and closely wedged between two ovarian tumours. The left ovarium filled completely that side of the cavity of the pelvis, and rose one and a half or two inches above the brim having the fallopian tube and broad ligament expanded over it. The fundus of the vesica urinaria, which had been emptied just before the patient was placed on the table, was close behind the symphysis pubis, and appeared to occupy but little space. The right ovarium, on the right side of the hydatid portion, was elevated about four inches above the brim of the pelvis; and was, to my surprise, firmly attached all round the brim and sides, from the crest of the pubis to the projection of the sacrum. From the sulcus formed between the hydatid portion, and that covered by the peritoneum, and strongly attached to it, a bright red fillet or band of arteries, about five-eighths of an inch wide, and six inches long, and resembling, in regularity of arrangement and size, the texture of a gum elastic suspender, extended obliquely across the median line of the body, and was attached to the omentum high up in the left hypochondriac region. From the highly injected appearance of the investing membrane of this fillet, I am induced to think that it was the seat of the pain in the above region of the abdomen, so frequently complained of by the patient; which pain had suddenly returned two days before the operation, and still existed at the time of it. As these arteries, from their size and number, seemed to afford the principal supply of blood to the tumour, a single leather ligature was thrown around the whole, tied firmly and cut off close to the knot. They were then divided within half an inch of the tumour. I next tried to introduce my fingers between the tumour and the brim of the pelvis, but this was rendered impossible by the firmness of the bands above alluded to. It was now found necessary to extend the incision through the skin, fascia and peritoneum down to the symphysis pubis, to facilitate the dissection of the anterior part of the tumour. I then commenced near the crest of the pubis, and by means of the scalpel, probe-pointed bistoury, and the fingers, cautiously separated the adhesions two-thirds of the way round the right side of the pelvis. At this point the bands were particularly firm, and in dividing them an artery of considerable size was cut. This was tied with a leather ligature, and cut off close. It was at this stage of the operation, that in raising a flap of thickened peritoneum, I discovered beneath it the proper, or albugineous coat of the ovarian tumour, and the whole character of the disease was disclosed. In almost all ovarian tumours, we find them covered with a complete peritoneal investment. In this case it appeared, that the inferior portion of the right ovarium first became diseased and expanded, and in doing so, after having separated the folds of the peritoneum composing the broad ligament, dipped down into the pelvis, and gradually filled it on the right side. As the tumour increased, it raised up the peritoneum lining that side to the level of the brim, and there it became firmly attached. But one artery passed into the tumour in that direction. After this was cut and tied, and the peculiar relations of the tumour were exposed, one sweep of the bistoury sufficed to clear it from its posterior connections, and after breaking up the cellular attachments in the basin of the pelvis with my fingers, I raised the whole out of its bed. That portion of the tumour above the brim, overlapped the iliac artery, and I felt it pulsating strongly when I separated the tumour.

Previous to this time, the small intestines were protruding through the external wound, and interfered with the dissection. Drs. Neff and Parry, had been requested to make pressure upon the sides of the abdomen, with the intention of confining them under the relaxed integuments; but we soon found that this pressure only occasioned a more obstinate protusion; and during the remainder of the operation, they were easily restrained by my brother, who supported them within the cavity, in the palm of his hand, in the upper angle of the external wound. It was, therefore, unnecessary to use the soft flannel envelopes, wrung out of warm water, previously provided. While engaged in doing this, the extremity of his fingers rested upon the aorta. The benefit of the previous treatment was strongly exemplified in the collapsed appearance of the intestines. They lay flat within the abdomen, and quite free from flatulent distension. Their peritoneal coat was of a pale pink colour, and slightly injected.

So great was the development of this ovarian tumour within the pelvis, and so closely adherent to the uterus, that considerable traction had to be made on one side, while my brother drew the uterus in the opposite direction, before I could pass a common suture needle, armed with a strong double ligature, through the broad ligament between them. The pedicle was then tied very lightly above and below. At the moment of tying the upper one, or that embracing the fallopian tube, the patient complained of considerable pain. Additional force was now necessary to draw the tumour from the ligatures, so as to afford space between them to sever the ligament. It was much thickened and very vascular. The tumour was now removed on the right side. That formed by the left ovarium next required attention, and occasioned but little difficulty. It was covered throughout by peritoneum and was unadherent, except by its natural attachment. As before stated, it filled up completely the left side of the cavity of the pelvis, the uterus being jammed closely between the two. It rose about two inches above the brim. Passing the fingers of the right hand between the side of the pelvis and the tumour, it was readily slipped from its bed, and given to be held by the assistant. Another needle, armed with a double silk ligature, was then passed as before, and tied above and below. The patient complained of similar pain when the left fallopian tube was tied. The broad ligament was now divided and the tumour removed. The uterus, free from disease, remained standing upright in the pelvis and shorn of its natural attachments. The cavity of the pelvis, which contained some blood and more serum, (for very little hæmorrhage attended the operation,) was now quickly cleansed by a succession of *very soft* sponges, rapidly handed to me by careful assistants. This I considered preferable to turning her upon her face, as was done in some previous operations, for the purpose of evacuating the fluids. Deeming it most advisable to remove the leather ligature around the omental arteries, as my leather ligatures had not been satisfactorily prepared, I did so, and replaced it by a silk one—one end of it was cut off close and the other brought out at the lower orifice of the wound. No hæmorrhage followed the removal of the ligature, and on squeezing the extremity of the fillet of arteries, several circular coagula escaped from their mouths. The edges of the whole external wound were now carefully approximated and secured by seven *hair-lip* sutures—separated about an inch from each other. These sutures were made by passing common small sized sewing needles, held in a "*porte-aiguille*," deeply through the parietes, but exterior to the fascia and peritoneum. The intervening spaces were covered with adhesive strips—the ligatures, five in number—the arterial one being marked by a double knot—were brought out below, turned over on the left side, and secured by a strip of adhesive plaster. Over these strips a folded portion of patent lint was placed; a folded napkin as a compress; and the whole secured by a broad and firm bandage, carefully pinned. The wet clothes were replaced by dry ones, and the patient carried to bed and placed on her back, with her head and shoulders slightly raised, and the limbs extended. Twenty drops of elixir of opium were then given to her. The operation lasted about 45 minutes—15 of which were spent in removing the water through the canula. By far the largest portion of time was occupied in separating the large tumour from the brim and sides of the pelvis. The operation was not interfered with by the occurrence of any unfavourable symptoms, neither nausea nor vomiting occurred then, nor subsequently. There were was some eructations of wind just before removing her from the table. The pulse, which was 104 at the commencement, was precisely 100 at the conclusion, with some, and but slight, diminution of force. During the whole of this trying scene the patient evinced the utmost composure and fortitude; replying in a firm tone to such questions as were necessary; and expressed herself with cheerfulness and gratitude when informed that it was concluded."

Nothwithstanding the great severity of the operation, and extensive exposure of the abdominal contents, the patient recovered; being relieved both of the diseased ovaria and the ascites. On the 22d of July, Dr. Atlee ceased visiting his patient "with any regularity;" on the 26th of September the last ligature came away, and the fistulous opening immediately healed; on the 21st of October, the patient, after an absence of six weeks returned to town, much improved in flesh and strength, and apparently restored to perfect health. The treatment of the case, from the time that Dr. Atlee first took charge of the patient, until her recovery from the perilous operation, appears to have been highly judicious, and the operation skilfully performed. In our next number we shall endeavour to find space for a short notice of the case of Mr. Southam of Manchester, operated on in March last, and that of Mr. Walne, in September—being his third case.

---

### WOUND OF THE AORTA AND PERICARDIUM.

The following case is interesting in a medico-legal point of view:—A Spanish refugee was struck by one of his companions with a knife in the back. The blade broke at a little distance from the skin. The patient walked to the hospital, where he died two hours after. At the *post-mortem* examination, it was found that the knife had penetrated between the seventh and eighth dorsal spines, that it had cut or broken a portion of one of these processes, crossed obliquely the vertebral canal, traversed the body of the vertebra from below, and a little to the right side of the centre, and then wounded the aorta below its arch. The pericardium was divided to the extent of five millimetres; it contained three grammes of blood. The pleuræ, but more especially the left, were filled with a considerable quantity. The spinal cord was not affected.—*Dublin Journal*, from *Bull. de Thér.*

